# Not all are the same – the power of registries in defining genotype-phenotype relationships in primary ciliary dyskinesia

**DOI:** 10.1183/13993003.01026-2024

**Published:** 2024-08-08

**Authors:** Amjad Horani, Pleasantine Mill

**Affiliations:** 1 Washington University School of Medicine, Division of Pediatric Allergy, Immunology, and Pulmonary Medicine, St Louis, MO, USA; 2 MRC Human Genetics Unit, Institute for Genetics and Cancer, University of Edinburgh, Edinburgh, UK

Steve Jobs once said ‘You can’t connect the dots looking forward; you can only connect them looking backwards. So you have to trust that the dots will somehow connect in your future.’ This holds true especially when dealing with rare conditions. Patterns often only emerge when you step back from individual patients and clinics and look over large multicentre datasets. Clinical registries are set up with the anticipation of finding patterns that can shed light onto disease processes, prognosis, and differences between patient groups. Registries require a significant upfront investment and time to set-up and more so to scale up to capture the necessary number of patients to see patterns emerge. The study led by Raidt et al[1] in this issue of the *European Respiratory Journal* exemplifies the power and importance of such large multiscale clinical datasets with genotypes, in stratifying patients moving forward.

Primary ciliary dyskinesia (PCD) is a rare genetic condition caused by dysfunction of motile cilia. Multiple motile cilia are small hair-like structures that line the respiratory tract, brain ventricles and reproductive tracts[2, 3]. Single motile cilia are also found on the transient node structure during early embryogenesis which functions in the establishment of left-right body asymmetry, as well as the sperm flagellum. The coordinated movement of motile cilia creates an effective fluid flow, such as is necessary for mucociliary clearance in the airways. Loss of this coordinated movement leads to significant lung disease, recurrent otitis media, conductive hearing loss, rhinosinusitis, hydrocephaly, and situs abnormalities including congenital heart defects. PCD is caused by variants in over 53 genes, yet common practice has treated all variants as similar[4]. However, there is mounting evidence reporting differences in disease severity in groups of patients carrying variants in specific genes[5–7] as well as different variants within the same gene[8]. These differences raise questions on the extent of genotype-phenotype relationships, and whether PCD should be viewed as a spectrum of disease severities.

The study by Raidt et al[1] demonstrates why a collaborative and concerted effort across many nations is necessary in caring for patients with rare conditions. It brings together 19 countries within the European Reference Network on respiratory diseases (ERN LUNG), in Europe, the Middle East, and South America to create the first international PCD Registry. Their data surveyed a staggering 1,236 patients with PCD, with confirmed pathogenic variants in 46 PCD genes, representing the largest and most encompassing PCD dataset to date. Genetic data was tied to clinical measurements like body mass index, laterality defects, and longitudinal lung function measurements (e.g. forced expiratory volume in 1 second, FEV_1_). Once all these data points are analysed, one can start to see patterns and important connections emerge. Not all genes are the same, and not all places are affected equally in PCD.

While some of this has been reported in smaller studies for a subset of genes, more data across more genotypes offer a more comprehensive clinical overview of the genetic landscape of PCD[6, 7, 9–11]. Here, Raidt et al[1] clearly demonstrate that individuals with variants in *CCDC39* and *CCDC40*, causing microtubular disorganisation, as well as *CCNO* and *MCIDAS* which reduce the number of motile cilia, have worse outcomes. These patients have a significantly worse lung disease (FEV1 z-score: -2.96, -2.96, -3.26, -4.36 respectively) than that of the whole patient cohort (FEV1 z-score: -1.66). By the same token, patients with variants in *DNAH11* and *ODAD1* have better lung functions (-0.83 and -0.85, respectively). 528 individuals (43% of individuals in the cohort) had multiple data points collected over time. Based on this data, individuals with *CCNO, CCDC39*, and *CCDC40* have an accelerated decline in lung function. However, across all PCD gene data sets here, we can see clearly that PCD is never a benign disease and has a significant clinical burden. Further, this finding highlights the urgency for genotype-led stratification of patients with PCD in terms of prognosis, management and recruitment into clinical trials. It is important to note, that 16/46 (35%) of genes had five or less patients in this cohort, which limits the precision of conclusions drawn. Recruiting more patients of more genotypes will be key to extending the power of this registry.

While progressive airway disease is a unifying feature of all PCD patients, the other non-respiratory features associated with PCD have been more difficult to quantify. This could be due to pleiotropy (a phenomenon in which a single gene affects two or more seemingly unrelated phenotypic traits) and/or penetrance (proportion of individuals with a particular genotype who express the associated phenotype). *Situs inversus* has been a hallmark of PCD since it was first described by Kartagener, affecting ~50% of individuals[12]. The frequency of other forms of laterality defects (such as heterotaxy and other congenital heart conditions) is less clear[13]. Raidt et al have revisited laterality defects in this large cohort and report that 55% of genetically confirmed PCD patients have normal laterality (*situs solitus*), while 39% have *situs inversus totalis*, and 3% have heterotaxy (*situs ambiguus)*. Importantly, when broken down by genes, laterality defects were significantly higher in variants associated with ciliary ultrastructural defects (51%) versus the rest of the PCD cohort (18%). Moreover, no laterality defects were reported in patients carrying variants in 17/46 genes (37% of PCD genes), the majority of which were associated with the central pair and radial spoke apparatus. This gene-based filter can help prioritise patients who should have cardiac evaluation and other imaging-based scans to rule out situs defects, where a range of heterotaxy features can be observed from striking to subtle[14].

Motile cilia in different organs may also have different compositions and altered waveforms, which in turn can affect non-respiratory PCD phenotypes[2, 3]. In the brain and spinal cord, motile cilia are essential for cerebrospinal fluid flow, and may play key functions in the development of the neuronal circuitry[15–17]. Hydrocephalus is a rare phenotype, reported in only 1.6% of PCD patients[18], that is increasingly recognized to be common in patients with specific PCD variants (*TUBB4B, CCNO, FOXJ1, MCIDAS*)[19–23], likely reflecting defects in ependymal or choroid plexus cilia function. Moreover, the magnitude of learning disabilities and neurodevelopmental disorders in PCD is not well characterised, though often reported on by parents[24]. Teasing out this data will require a concerted effort and massive data collection on the scale presented here. Other issues related to cilia function such as fertility issues are also often not well defined. Understanding this data will allow better prioritisation of access to and management of reproductive services for subsets of patients more prominently affected.

Finally, this report by Raidt et al highlights the differences in diagnostic approaches in different regions, as no globally integrated or standardised diagnostic guideline exists[4, 25]. Part of these differences may be attributed to different guideline recommendations from the major respiratory societies (American[26] versus European[27]), but also to access to resources[28]. Within different regions, different founder mutations may exist, with different genes most commonly affected and sometimes even resulting in atypical presentations[4, 10, 29]. Large international registries like this are key to be able to recognise and diagnose more patients globally.

There are different ways to look at dots, or patient data, for connections. The current study focused on countries as a means to parse common genes and variants in PCD which helps demarcate institutional elements affecting PCD diagnosis. But borders don’t define populations and diversity within groups can vary hugely in terms of race and ethnicity. Defining clinical presentation based on ethnicity is critical to identifying patients within minority groups or individuals from diverse ethnic backgrounds. Although a sensitive subject and unconscious bias may exist, it is an important aspect as it informs on possible missing patient cohorts. A recent study suggested that the prevalence of PCD is much higher in individuals of African ancestry[30], yet those patients are not identified readily in our clinics, suggesting a diagnostic or referral bias[29]. As a result, many PCD longitudinal studies do not have enough ethnic diversity in their cohorts resulting in bias defining the molecular and clinical features within this condition. Larger studies like this registry can capture ethnic ancestry and allow comparison between different groups. The scale and multipartner buy-in of such registries are critical for shaping diagnostic and counselling strategies for diverse populations.

Despite the limitations, this study represents a huge leap for the field in understanding genotype-phenotype relations, which will profoundly impact our approach to patient diagnosis, prognosis and possibly clinical trial design. We need more power - by coming together; combining registries across countries, regions, and continents, will lead to more dots and more diversity. In turn, this will allow us to see the bigger picture. A big picture for the benefit of all patients with PCD.

The authors of this editorial call on the major regional PCD societies to join forces. As Raidt et al[1] have shown, it is better together for not all PCD is the same and no one can see the whole picture alone.

## Figures and Tables

**Figure 1:**
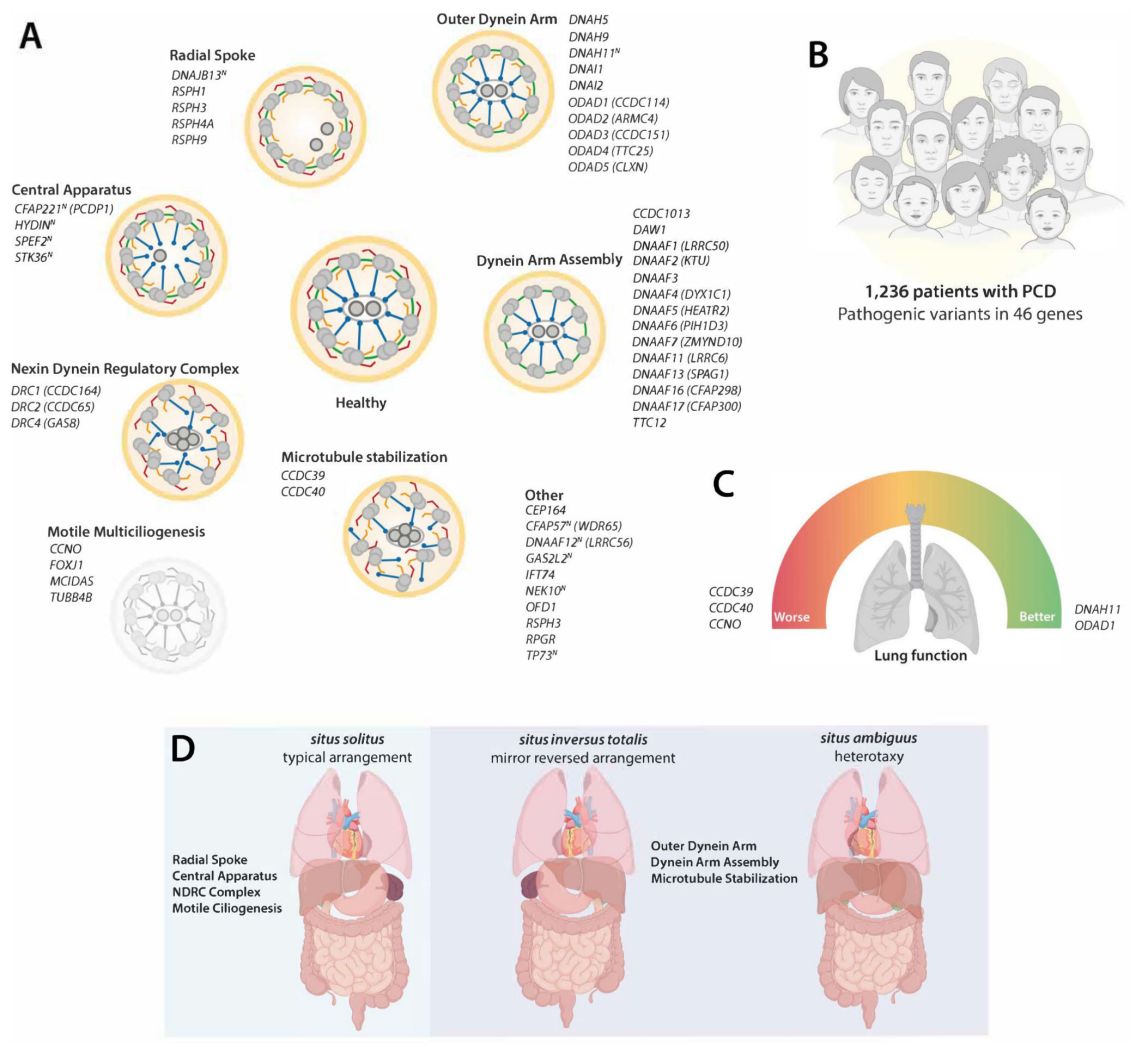
Revealing genotype-phenotype relationships in PCD. (A) Schematic of motile cilia axoneme cross-section representing ultrastructure required for motility (healthy donor) compared to groups based on structure affected in patients with PCD. 52 PCD genes are listed in italics, with superscript (^*N*^) representing near normal ultrastructure. Outer dynein arm (red); inner dynein arm (orange); nexin dynein regulatory complex (green); radial spokes (blue) and central pair apparatus (dark grey). (B) Study overview with number of patients with PCD included from the ERN-LUNG International PCD registry, who carry pathogenic variants in 46 genes. (C) Analysis of large cohorts reveal genotype-phenotype relationships at a gene-level, such as for lung function (FEV_1_) both as individual data points and longitudinally. Patients with variants in *CCDC39, CCDC40* and *CCNO* have significantly worse lung function (red dial, left) and decline faster than patients with variants in *DNAH11* or *ODAD1* (green dial, right). (D) In contrast, situs defects are not observed in patients with PCD who have pathogenic variants affecting genes required for motile multiciliogenesis, radial spokes, central apparatus or nexin dynein regulatory complex (blue box, left), in contrast to those affecting formation or function of dynein arms which do *show situs inversus totalis* or heterotaxy (purple box, right). Elements of figure panels created with BioRender.
